# Lymph Node Micrometastases are Associated with Worse Survival in Patients with Otherwise Node-Negative Hilar Cholangiocarcinoma

**DOI:** 10.1245/s10434-015-4723-9

**Published:** 2015-07-16

**Authors:** Hendrik T. J. Mantel, Jim K. Wiggers, Joanne Verheij, Jan J. Doff, Egbert Sieders, Thomas M. van Gulik, Annette S. H. Gouw, Robert J. Porte

**Affiliations:** Department of Hepato-Pancreatico-Biliary Surgery and Liver Transplantation, University Medical Center Groningen, University of Groningen, Groningen, The Netherlands; Department of Hepato-Pancreatico-Biliary Surgery, Academic Medical Center, University of Amsterdam, Amsterdam, The Netherlands; Department of Pathology, Academic Medical Center, University of Amsterdam, Amsterdam, The Netherlands; Department of Pathology, University Medical Center Groningen, University of Groningen, Groningen, The Netherlands

## Abstract

**Background:**

Lymph node metastases on routine histology are a strong negative predictor for survival after resection of hilar cholangiocarcinoma. Additional immunohistochemistry can detect lymph node micrometastases in patients who are otherwise node negative, but the prognostic value is unsure. The objective of this study was to assess the effect on survival of immunohistochemically detected lymph node micrometastases in patients with node-negative (pN0) hilar cholangiocarcinoma on routine histology.

**Methods:**

Between 1990 and 2010, a total of 146 patients underwent curative-intent resection of hilar cholangiocarcinoma with regional lymphadenectomy at two university medical centers in the Netherlands. Ninety-one patients (62 %) without lymph node metastases at routine histology were included. Micrometastases were identified by multiple sectioning of all lymph nodes and additional immunostaining with an antibody against cytokeratin 19 (K19). The association with overall survival was assessed in univariable and multivariable analysis. Median follow-up was 48 months.

**Results:**

Micrometastases were identified in 16 (5 %) of 324 lymph nodes, corresponding to 11 (12 %) of 91 patients. There were no differences in clinical variables between K19 lymph node-positive and -negative patients. Five-year survival rates in patients with lymph node micrometastases were significantly lower compared to patients without micrometastases (27 vs. 54 %, *P* = 0.01). Multivariable analysis confirmed micrometastases as an independent prognostic factor for survival (adjusted Hazard ratio 2.4, *P* = 0.02).

**Conclusions:**

Lymph node micrometastases are associated with worse survival after resection of hilar cholangiocarcinoma. Immunohistochemical detection of lymph node micrometastases leads to better staging of patients who were initially diagnosed with node-negative (pN0) hilar cholangiocarcinoma on routine histology.

Hilar cholangiocarcinoma, also known as Klatskin tumor, is a malignancy originating from the biliary epithelium at the confluence of the left and right hepatic duct.^1^ Cure can only be achieved by complete surgical resection of the tumor, consisting of extrahepatic bile duct resection with a partial hepatectomy, lymphadenectomy of the hepatoduodenal ligament, and occasionally portal vein or hepatic artery resection.^2^–^4^ Despite recent improvements in surgical techniques, the prognosis after curative resection remains poor, with reported 5-year survival rates below 40 %.^2^,^4^–^6^

Regional lymph node status is known to be an important predictor of survival after resection.^2^,^4^,^5^,^7^,^8^ However, even patients without lymph node metastases at postoperative pathologic examination frequently develop tumor recurrence. One possible explanation is the presence of micrometastases in the lymph nodes, resulting in understaging of the disease.

Conventional routine histologic examination of the resected specimen includes single sectioning of the lymph nodes with hematoxylin and eosin (H&E) staining. This procedure may underestimate the incidence of nodal metastases. Additional immunohistochemical staining with cytokeratin antibodies may facilitate the detection of small tumor deposits in lymph nodes (micrometastases). In recent years, a number of studies have shown the clinical significance of immunohistochemically detected lymph node micrometastases in a variety of tumors, including those of breast, lung, esophagus, stomach, colon, and gallbladder.^9^–^21^ Regarding hilar cholangiocarcinoma, contradicting reports have been published.^22^–^24^

This study was undertaken to further investigate the significance of multiple lymph node sectioning and additional immunohistochemical detection of micrometastases on the survival of patients who were initially classified as having lymph node-negative (pN0) hilar cholangiocarcinoma on the basis of conventional histologic examination.

## Methods

### Patients and Operation

Between January 1990 and July 2010, a total of 146 patients underwent a curative-intent resection and systematic lymph node dissection of the hepatoduodenal ligament for hilar cholangiocarcinoma at two university medical centers in the Netherlands. Fifty-six patients were treated at the University Medical Center Groningen (UMCG), and 90 were treated at the Academic Medical Center Amsterdam (AMC). In 91 (62 %) of these patients, no lymph node metastases were detected by conventional histologic examination (H&E staining) of the surgical specimen. There were 49 male and 42 female patients. Mean age was 62 ± 9 years (range 36–78 years).

All patients underwent extrahepatic bile duct resection with lymphadenectomy of the liver hilum, in most cases in combination with (extended) hemihepatectomy. Standard regional lymph node dissection consisted of an exploration of the hepatoduodenal ligament and skeletonization of the portal vein and hepatic artery after the distal common bile duct was cut at the level of the pancreatic head and dissected free. In accordance with the American Joint Committee on Cancer (AJCC; 7th edition), lymph nodes at the following locations were defined as regional: along the cystic duct, common bile duct, proper hepatic artery, and portal vein.^25^ Routine dissection of more distant lymph nodes was not performed. However, suspected distant lymph nodes (N2) were sampled via biopsy, and the operation was aborted if intraoperative frozen section analysis of these samples showed tumor cells.

The majority of patients (91 %) underwent a concomitant partial hepatectomy. Left hemihepatectomy (either extended or not) was performed in 38 patients (42 %) and (extended) right hemihepatectomy was performed in 35 patients (38 %). Ten patients (11 %) underwent only concomitant segment 4 resection. Portal vein resection and reconstruction was undertaken in 18 patients (20 %), and arterial resection and reconstruction was undertaken in 2 patients (2 %). One patient underwent combined arterial and portal venous reconstruction. Tumors were staged according to the tumor, node, metastasis classification system as proposed by the AJCC.^25^ A total of 324 lymph nodes were retrieved from 91 surgical specimens. On all lymph nodes, additional immunohistochemical staining of cytokeratin 19 (K19) was performed.

None of the patients in either institute received postoperative chemotherapy or radiotherapy because this is not recommended in the Dutch guidelines for bile duct carcinoma.

### Immunohistochemistry

Formalin-fixed, paraffin-embedded tissue blocks of the lymph nodes were retrieved from the tissue archives of the departments of pathology in the AMC and UMCG. Four new levels of each lymph node with a distance of 250 µm between the levels were investigated. Two 4 µm thick sections of each level were cut serially. One section was stained with H&E and one with an antibody against K19 (dilation 1:100, clone RCK108; Dako, Glostrup, Denmark). All staining procedures were performed in the UMCG. The K19 staining was performed using a Ventana Benchmark Ultra automated stainer (Ventana Medical Systems, Tucson, AZ, USA) after pretreatment with protease.

Micrometastases were defined as cells detected by immunostaining with morphologic features of adenocarcinoma. H&E and K19 immunolabeled slides were investigated by two experienced pathologists (ASHG and JJD) blinded to the demographic and clinicopathologic features of patients.

### End Points

The effect of micrometastases was assessed in terms of patient survival and time to recurrence. Patients who died during postoperative hospital admission were excluded from the analyses because they were unlikely to have died from recurrent disease. Patient survival was determined from the time of surgery to the time of death or most recent follow-up (March 17, 2014). No patient was lost to follow-up. The median follow-up among survivors was 52 months (range 8 months to 20 years).

Recurrences were defined as any new lesion on imaging that was highly suspicious for recurrence of hilar cholangiocarcinoma. Pathologic confirmation was often obtained but was not required. Recurrences at the liver resection margin, distal bile duct remnant, hepaticojejunostomy, or elsewhere in the liver hilum were classified as local recurrences. All other recurrences were classified as distant. Time to recurrence was measured from the time of surgery to the time of the first recurrence. Patients who had no observed recurrence were censored at the time of last follow-up, and patients who died from other causes before developing a recurrence were censored at the time of death.

### Statistical Analysis

Patient data and baseline characteristics were retrospectively collected in a database, and statistical analyses were carried out by SPSS Statistics software (IBM, Armonk, New York, USA). Continuous variables were expressed as mean ± SD. Categorical variables were expressed as numbers and percentages. Comparison of means was performed with Student’s *t* test for independent samples. Comparison of categorical variables was performed with the *χ*^2^ test or Fisher’s exact probability test. Univariable analyses were conducted for patient survival and time to recurrence by Kaplan–Meier estimates of survival probabilities and the log-rank test for comparisons. A Cox proportional hazard regression model was used to analyze associations with patient survival in multivariable analysis, including all factors with a *P* value of less than 0.10 in univariable analysis. Because AJCC pT1 stage was considered a potential confounder, we a priori included this variable in the Cox model. *P* values were two sided, and values of less than 0.05 were considered statistically significant.

## Results

**Fig. 1:**
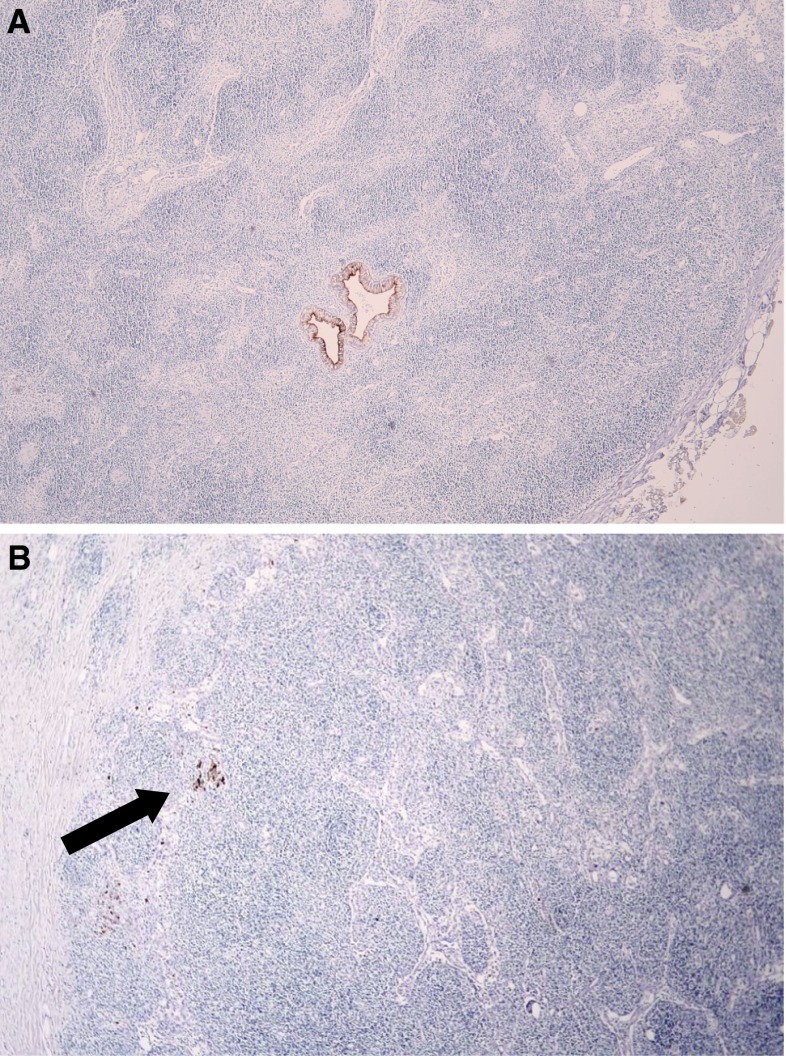
Cytokeratin 19-positive metastatic tumor cells in regional lymph nodes from resected hilar cholangiocarcinoma specimens. **a** Micrometastases consisting of two small tumor glands (original magnification, ×4). **b** Micrometastases consisting of single tumor cells (*arrow;* original magnification, ×4)

Micrometastases were detected in 16 (5 %) of 324 lymph nodes and in 11 (12 %) of 91 patients who were initially considered lymph node negative by conventional histologic examination (Fig. 1). The K19 labeling clearly highlighted the adenocarcinoma foci and confirmed their biliary origin.

Postoperative death during hospital admission (in-hospital mortality) occurred in 11 (12 %) of 91 patients, who were subsequently excluded from the survival analyses. In-hospital mortality occurred only in the group without micrometastases (range 8–80 days). Clinicopathologic details of the remaining 80 patients with and without lymph node micrometastases are shown in Table 1. There were no statistically significant differences between the two groups. During postoperative follow-up, 49 (61 %) of 80 patients died. At the end of follow-up, 31 patients were alive, two of whom were diagnosed with disease recurrence.

**Table 1:** Clinicopathologic characteristics of patients after curative-intent resection for hilar cholangiocarcinoma with and without lymph node micrometastases

Characteristic	Lymph node micrometastases		*P*
	Absent (*n* = 69)	Present (*n* = 11)	
Mean age in years (±SD)	61 ± 10	59 ± 6	0.40
Gender male/female	37/32	5/6	0.75
Type of hepatectomy			
Left	25	4	0.90
Extended left	6	0	
Right	5	1	
Extended right	17	4	
Only segment 4 resection	9	1	
No liver resection	7	1	
pT stage^a^			
pT1	14	0	0.48
pT2a	32	5	
pT2b	12	3	
pT3	3	1	
pT4	8	2	
Microscopic resection margin positive	20 (29 %)	4 (36 %)	0.73
Perineural invasion positive	39 (57 %)	8 (73 %)	0.31
Complication rate grade III or IV^b^	24 (35 %)	4 (36 %)	0.92
Mean no. of dissected lymph nodes per patient	4	5	0.18

Patients with in-hospital mortality (*n* = 11) were excluded from analysis

^a^According to American Joint Committee on Cancer staging manual, 7th edition

^b^According to Clavien-Dindo classification of surgical complications. Grade V (in-hospital mortality) was excluded

## Survival Analysis

**Fig. 2:**
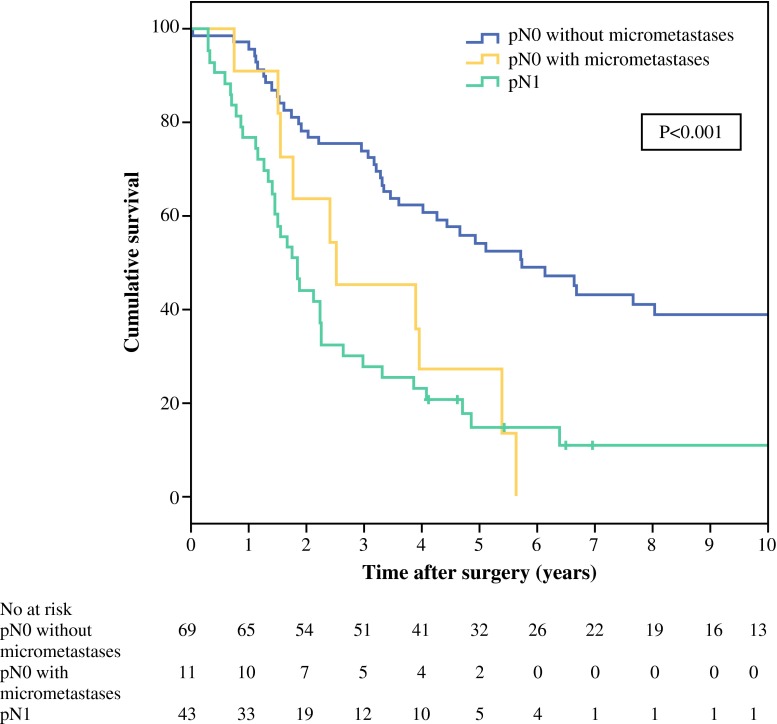
Survival after resection for hilar cholangiocarcinoma according to lymph node status. Patients with in-hospital mortality were excluded from analysis. pN0 without micrometastases versus pN0 with micrometastases: *P* = 0.01 (log-rank test). pN0 with micrometastases versus pN1: *P* = 0.54 (log-rank test)

The survival rates for patients were calculated according to lymph node status. We defined three groups: patients with lymph node (macro)metastases detected at routine H&E examination (pN1); patients with lymph node micrometastases detected with multiple sectioning and K19 staining (pN0 with micrometastases); and patients without lymph node micrometastases (pN0 without micrometastases). Five-year survival rates in patients without lymph node micrometastases were significantly higher compared to the other groups (*P* < 0.001) (Fig. 2). There was a significant difference in 5-year survival between patients with and without micrometastases (27 vs. 54 %, *P* = 0.01), but not between patients with micro- and macrometastases (27 vs. 15 %, *P* = 0.54).

## Recurrence

**Fig. 3:**
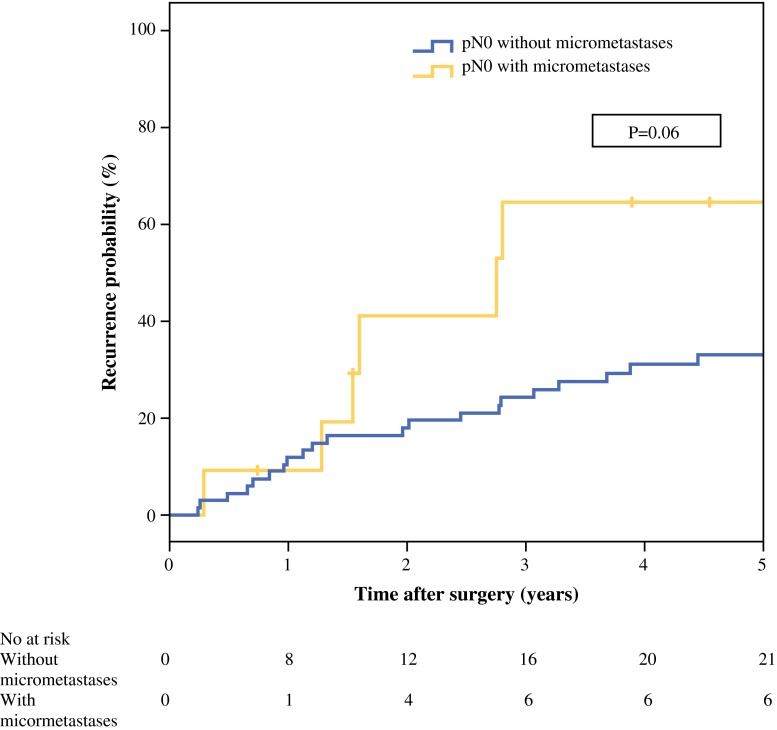
Cumulative probability of recurrence after resection for hilar cholangiocarcinoma in patients classified as pN0 based on routine histologic examination, with or without lymph node micrometastases on subsequent immunohistochemistry. Patients with in-hospital mortality (*n* = 11) were excluded from analysis; *P* = 0.06 (log-rank test)

During the study period, 30 (38 %) of 80 patients developed disease recurrence (local recurrence in 21 patients and distant metastases in 9 patients). Figure 3 presents the estimated cumulative probability of recurrence over time according to the presence or absence of micrometastases. At 5-year follow-up, the estimated probability of recurrence was 65 % in the group with micrometastases, versus 33 % in patients without micrometastases (*P* = 0.06).

Next, the relationship between lymph node micrometastases and pattern of recurrence was analyzed. There was a twofold higher percentage of distant site recurrences in the group with micrometastases compared to the group without micrometastases (18 vs. 9 %), but this did not reach statistical significance (*P* = 0.40).

## Analysis of Prognostic Factors in Patients with pN0 Disease

Prognostic factors for survival were identified using univariable analyses (Table 2). Two variables—microscopic resection margin status (*P* = 0.09) and lymph node micrometastases (*P* = 0.01)—were identified as predictors for survival. These two variables were included in a multivariable Cox regression analysis, together with AJCC pT stage, because this was a priori considered a possible confounding variable. Only the presence of micrometastases was identified as an independent predictive factor for survival in patients with pN0 hilar cholangiocarcinoma (Hazard ratio 2.43, 95 % confidence interval 1.16–5.10) (Tables 2).

**Table 2:** Univariable analysis of survival in patients with hilar cholangiocarcinoma classified as pN0 based on routine histologic examination

Variable	No. patients	5-year survival (%)	*P*
Univariable analysis			
Age			
<60 year	30 (38 %)	43	0.38
≥60 year	50 (62 %)	55	
Sex			
Male	42 (53 %)	49	0.71
Female	38 (47 %)	52	
Type of hepatectomy^a^			
Left	45 (63 %)	50	0.40
Right	27 (37 %)	40	
pT stage^b^			
T1	14 (17 %)	71	0.37
T2a and 2b	52 (66 %)	47	
T3 and 4	14 (17 %)	41	
Perineural invasion			
Negative	33 (41 %)	47	0.60
Positive	47 (59 %)	53	
Lymph node micrometastases			
Negative	69 (86 %)	54	0.01
Positive	11 (14 %)	27	
Microscopic resection margin status			
Negative	56 (70 %)	56	0.09
Positive	24 (30 %)	38	

Variable	Hazard ratio	95 % confidence interval	*P*
Multivariable analysis			
pT stage^b^			
T1	Reference category		
T2a and T2b	1.55	0.64–3.75	0.33
T3 and T4	1.25	0.42–3.76	0.69
Lymph node micrometastases	2.43	1.16–5.10	0.02
Microscopic resection margin status	1.54	0.85–2.80	0.16

Patients with in-hospital mortality were excluded from analysis

^a^Patients without concomitant liver resection (*n* = 8) were excluded from analysis

^b^According to American Joint Committee on Cancer staging manual, 7th edition

## Discussion

Lymph node metastases have been repeatedly identified as an important prognostic factor for survival after resection of hilar cholangiocarcinoma.^2^,^5^,^26^ This has also been the experience of the two centers participating in this study.^4^,^7^ The current study was undertaken to improve tumor staging by identifying lymph node micrometastases in the pN0 group. To this end, we applied rigorous multiple sectioning of the lymph node tissue blocks to achieve deeper levels, investigated several levels of each lymph node, and increased the sensitivity of tumor cell identification by K19 immunolabeling. This method is a slight modification of the histopathologic investigation of sentinel nodes in other types of cancer (e.g., breast cancer and melanoma). Using this technique, micrometastases were detected in 5 % of the collected lymph nodes, which related to 12 % of the 91 patients who were initially characterized as pN0 on the basis of conventional histologic examination of the surgical resection specimen with H&E staining. Survival in the group with lymph node micrometastases was significantly worse compared to patients without micrometastases and comparable to patients with pN1 disease. There was a trend toward earlier tumor recurrence in patients with micrometastases, although this did not reach statistical significance (Fig. 3). Remarkably, the presence of lymph node micrometastases was found to be a stronger predictor than the level of tumor invasion (T stage) and microscopic resection margin status (Table 2). The same phenomenon was previously observed in a study by Yonemori et al.^24^ A possible explanation is that lymph node micrometastases represent a more aggressive biologic behavior of the tumor, similar to lymph node metastases found on routine histology.

Only three previous studies, all from Japan, have addressed the effect of lymph node micrometastases on survival in hilar cholangiocarcinoma, and these studies have shown contradictory results.^22^–^24^ Yonemori et al. found lymph node micrometastases to be of influence on the survival in a heterogeneous group of 151 patients with biliary cancer (including gallbladder cancer, intrahepatic cholangiocarcinoma, and ampullary cancer).^24^ A subgroup analysis of patients with hilar bile duct cancer only revealed lymph node micrometastases in 4 % of the investigated nodes and in 22 (27 %) of 83 patients, but no significant impact on survival was found. Similarly, Tojima et al. detected lymph node micrometastases in 13 (1.4 %) of 954 examined lymph nodes, corresponding to 11 (24 %) of 45 patients; they also concluded that lymph node micrometastases had no effect on postoperative survival.^23^ In contrast, Taniguchi et al. found lymph node micrometastases in 14 (3.3 %) of 423 lymph nodes and 11 (39 %) of 28 patients; these authors reported a significantly lower survival rate in patients with lymph node micrometastases.^22^

The current study is the first Western series in which the incidence of lymph node micrometastases and its influence on survival was investigated and presents the largest cohort published to date, consisting of 91 pN0 patients. There are some differences between our series and those reported in literature.

First, we found micrometastases in 12 % of the patients who were initially considered node negative on routine histologic examination. This is slightly less than the 24–39 % reported in the three Japanese series.^22^–^24^ The number of serial sections that were cut from the archival tissue blocks can be an explanation for this difference. In our study, four additional sections were performed, with a distance of 250 µm between consecutive levels, compared to five to eight serial sections reported in the three Japanese studies.^22^–^24^ We believe that with our technique all tumor cell clusters in the lymph nodes were detected. Some authors state that 200 µm should be the maximum distance between consecutive levels.^27^ When adopting this strategy, we should have investigated five instead of four levels, but it is questionable whether this extra level would have significantly increased the sensitivity of the procedure. To illustrate this, in the Japanese series, the percentage of patients in whom micrometastases were detected did not linearly rise with increased sectioning (24 % in the study with five serial sections, vs. 27 % in the study with eight serial sections).^23^,^24^ Recent studies of cancer of the breast, lung, stomach, and colorectum have classified metastatic tumor cells by size into micrometastases (>0.2 mm) and isolated tumor cells (≤0.2 mm).^23^,^24^,^28^–^31^ In our series, this type of further differentiation was not possible because in all cases metastatic tumor cells were larger than 0.2 mm in size (between 0.2 and 0.4 mm). Increased sectioning would probably have resulted in increased detection of isolated tumor cells. However, the significance of isolated tumor cells remains a subject of debate, even in breast cancer, where this issue has been studied more than in any other type of cancer.^32^

Second, lymphadenectomy in our population resulted in a mean of 4 lymph nodes per patient (range 1–16), which is less than the 10–20 nodes per patient reported in the Japanese series.^22^–^24^ However, this is in line with other Western series and in accordance with the AJCC/International Union Against Cancer guidelines, which recommend that at least 3 lymph nodes should be collected for adequate staging of hilar cholangiocarcinoma.^33^,^34^ The difference in number of collected lymph nodes between our study and the Japanese series can be explained by the variation in extent of the lymphadenectomy. In contrast to the Japanese experience, it is not routine surgical practice in the Netherlands to perform standard removal of N2 nodes (para-aortic, caval vein, and superior mesenteric). An N2 node is only removed when macroscopically suspicious and is then sent for intraoperative frozen sections analysis. The surgical procedure is aborted when frozen section analysis shows metastatic tumor cells.

The in-hospital mortality rate in the present study was 15 % for the entire group of patients and 12 % in the group with pN0 disease. Some series report a lower mortality rate, but in these series, patients who only underwent extrahepatic bile duct resection, without concomitant partial liver resection, were also included.^5^ Most of our patients (>90 %) underwent partial hepatectomy in addition to extrahepatic bile duct resection. Indeed, the postoperative mortality rate in our population is comparable to the mortality rate of 10–11 % described in other series in which partial hepatectomy was part of the surgical procedure.^34^–^36^

In the current study, micrometastases were detected by multiple sectioning of the lymph nodes together with K19 immunohistochemistry. In our opinion, application of both methods is important to detect micrometastases. In some cases, micrometastases were evident even without K19 staining (Fig. 1a) but would not have been found if additional sectioning had been omitted. In other cases, K19 labeling was necessary to highlight the small micrometastases.

In conclusion, this study shows that the presence of lymph node micrometastases in patients with otherwise node-negative hilar cholangiocarcinoma has a negative effect on survival. The technique of multiple lymph node sectioning together with K19 immunostaining results in improved staging of patients with hilar cholangiocarcinoma.
